# Total arch replacement for resection of a dedifferentiated liposarcoma of anterior mediastinal origin invading the aorta, left common carotid artery, and left subclavian artery: a case report

**DOI:** 10.1186/s44215-025-00191-9

**Published:** 2025-03-17

**Authors:** Hiroya Matabe, Takuya Nagashima, Yasuhiro Koga, Masuda Haruhiko, Ryo Izubuchi, Shota Yasuda, Keiji Uchida, Tetsukan Woo, Aya Saito

**Affiliations:** 1 Respiratory Disease Center, Yokohama City University Medical Center, 4-57 Urafune-Cho, Minami-Ku, Yokohama, Kanagawa 232-0024 Japan; 2 Cardiovascular Center, Yokohama City University Medical Center, 4-57 Urafune-Cho, Minami-Ku, Yokohama, Kanagawa 232-0024 Japan; 3 Department of Surgery, Yokohama City University, 3-9 Fukuura, Kanazawa-Ku, Yokohama, Kanagawa 236-0004 Japan

**Keywords:** Dedifferentiated liposarcomas, Mediastinal tumor, Total aortic replacement

## Abstract

**Background:**

Dedifferentiated liposarcomas of mediastinal origin are rare. They are prone to local recurrence and distant metastases; therefore, complete surgical resection is desirable and the most important prognostic factor. In this report, we describe a case of dedifferentiated liposarcoma involving the aortic arch, left common carotid artery, and left subclavian artery that was successfully resected via total arch replacement.

**Case presentation:**

A 78-year-old man presenting with hoarseness was diagnosed with a mediastinal tumor. After the examination, the tumor was suspected to be malignant, and the patient was referred to our hospital for surgery. We elected to remove the tumor using an artificial vessel replacement in the aortic arch. The surgery was performed, and the patient’s postoperative course was uneventful. The patient was discharged 26 days postoperatively. He is currently being followed up on an outpatient basis, with no signs of recurrence.

**Conclusions:**

We encountered a case of dedifferentiated liposarcoma involving the aortic arch, left common carotid artery, and left subclavian artery. Careful planning of the surgical procedures and complete resection of the tumor are important for successful outcomes in such cases.

## Background

Dedifferentiated liposarcomas predominantly occur in the retroperitoneum, inguinal region, and extremities; their occurrence in the mediastinum is rare. Complete tumor resection is the preferred treatment and the most important prognostic factor, as these tumors are locally invasive and can metastasize. To achieve complete resection, tumors should be removed along with the invaded organs. Herein, we describe a case of an anterior mediastinal tumor suspected of invading the aorta, successfully resected via total aortic replacement (TAR). The histological type was dedifferentiated liposarcoma.

## Case presentation

**Fig. 1:**
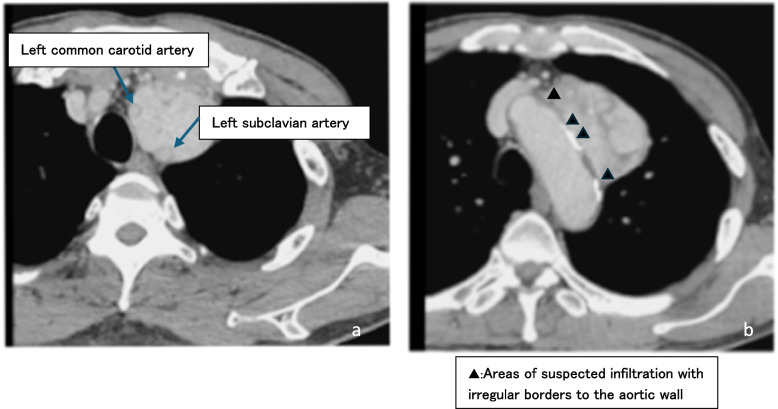
Enhanced chest computed tomography scan

A 78-year-old man presented to his doctor with hoarseness as the chief complaint and was referred to a nearby general hospital for consultation. Chest radiography revealed a mediastinal tumor, and the patient was then referred to our hospital. There was no elevation in tumor markers, interleukin-2, or anti-acetylcholine receptor antibodies. Contrast-enhanced computed tomography (CT) revealed an anterior mediastinal tumor measuring 59 mm × 40 mm × 84 mm, with heterogeneous contrast enhancement and internal calcifications (Fig. 1a). The tumor bordered the aortic arch and surrounded the left common carotid and subclavian arteries (Fig. 1b). It was suspected that the tumor had invaded the aortic arch, left common carotid artery, and left subclavian artery. Positron emission tomography revealed abnormal uptake of ^18^F-fluorodeoxyglucose in the tumor (maximum standardized uptake value = 6.7). There was no evidence of distant metastases. A thoracoscopic biopsy was performed. The MIB index was > 50%, and the possibility of malignancy could not be excluded. The patient was referred to our department for further treatment. On contrast-enhanced CT, the findings indicated difficulty in dissecting the tumor from the vessels. Indications for surgery are determined in consultation with the Department of Anesthesiology and Cardiovascular Surgery and the Cancer Board of the Department of Respiratory internal medicine and Radiology. As the result, the resection of the tumor could be achieved by performing TAR. Thus, we elected to remove the tumor surgically.

First, the left thoracic cavity was approached in the right lateral recumbent position using thoracoscopy. No tumor dissemination was observed. The tumor adhered to the aortic arch, making adhesion dissection impossible. The distal arch was dissected as far as possible, and the peritumoral pleura was dissected circumferentially. The left upper lung was partially resected because of tight adhesions. The left vagus nerve was resected because of preoperative paralysis caused by tumor invasion.

**Fig. 2:**
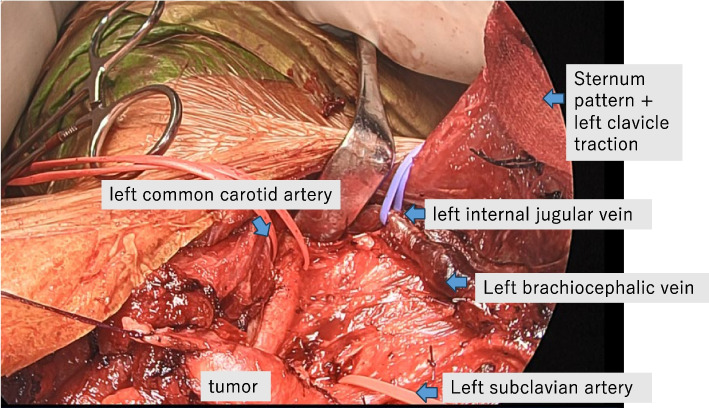
Approach to the tumor with transmanubrial approach

**Fig. 3:**
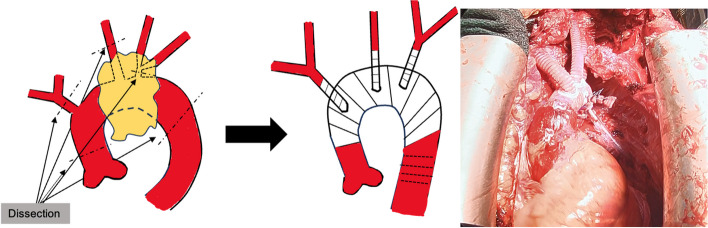
Performing total arch replacement to resect the tumor

**Fig. 4:**
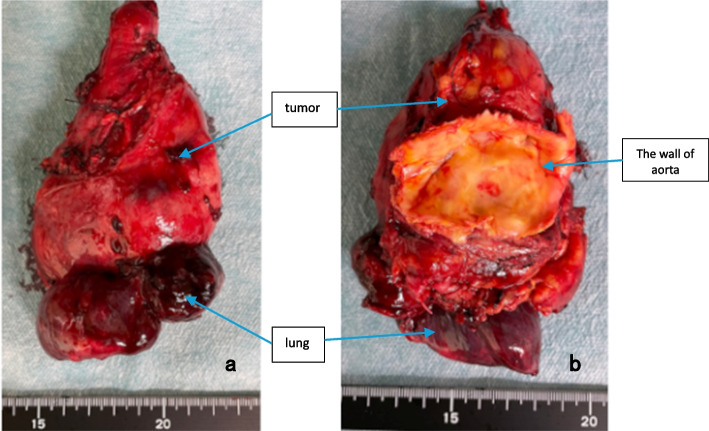
Tumor image

**Fig. 5:**
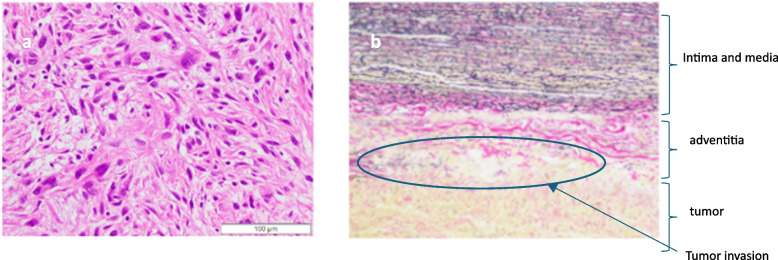
Tumor pathology

Second, to secure the left common carotid and left subclavian arteries more peripherally than in standard arch replacement, we used a transmanubrial approach (TMA). A median sternal incision was made, followed by an extended skin incision along the left sternocleidomastoid muscle. In addition, an approximately 10-cm skin incision was placed along the left second rib toward the left side. At the level of the first intercostal space, the sternum was transected to the left. The first rib was dissected near the sternal attachment point. The left side of the manubrium and the clavicular compartment were elevated while preserving the left sternoclavicular joint. This procedure secured the surgical field around the left subclavian vein. Adhesions between the tumor and surrounding tissues were dissected to the greatest extent possible, and various blood vessels were secured (Fig. 2). Tumor invasion into the innominate vein was resected using an autosuture device. Peripherally, the tumor was resected just after the bifurcation of the left common jugular vein and the left subclavian vein and centrally, just before the confluence of the superior vena cave. The left phrenic nerve was detected due to tumor invasion, whereas the right vagus and phrenic nerves were preserved. Subsequently, arterial cannulation was established by anastomosing an artificial vessel to the right femoral artery, and devascularization was performed in the superior and inferior vena cava to establish extracorporeal circulation. After cardiac arrest, the aorta was transected, and a 14-Fr cannula was inserted into the brachiocephalic artery and a 12-Fr cannula into the left common carotid artery for selective antegrade cerebral perfusion. The left subclavian artery was clamped because of confirmed contralateral circulation at the Willis arterial circle. The aorta arch was transected at Zone 3, and the tumor was resected with the aortic arch wall (Fig. 3). A vascular graft (Triplex® 20 m m 3 cm, Terumo, Japan) was guided into the descending thoracic aorta. Teflon felt was used to reinforce the outer aorta, and the graft was anastomosed to the aorta. The main graft (Triplex® 24 mm 4-Branch, Terumo, Japan) was anastomosed to an artificial vessel in the distal aorta. The ascending aorta was transected at its midpoint, and the main graft was anastomosed to it. The left subclavian artery, left carotid artery, and brachiocephalic artery were anastomosed to the artificial vessel in that order. After warming up, the patient was taken off cardiopulmonary bypass in sinus rhythm. Intraoperative blood loss was 666 mL. The tumor was a substantial grayish-white mass measuring 10.5 × 6.0 × 4.8 cm, tightly adherent to the aortic wall (Fig. 4a, b). It was mostly composed of dedifferentiated components, with some hyperdifferentiated areas (Fig. 5a). Immunostaining was positive for MDM2 and CDK4 expression. Moreover, a bone component was observed in part of the tumor, and invasion was observed in the adventitia of the aorta (Fig. 5b). The tumor was diagnosed as a dedifferentiated liposarcoma. The postoperative course was uneventful. The patient continued hoarseness but had no respiratory failure. The patient was discharged on postoperative day 26 with no recurrence for 6 months.

## Discussion and conclusions

Mediastinal liposarcomas occur in 0.1–0.75% of all mediastinal cases and are relatively rare [1, 2]. Dedifferentiated liposarcoma is defined as an undifferentiated sarcomatous growth adjacent to a well-differentiated liposarcoma within the same mass [3]. This highly malignant tumor has a local recurrence rate of 40% and distant metastases in 15–30% of cases, leading to a poor prognosis [4]. Surgical treatment is the first option when there are no distant metastases or unresectability factors. However, the recurrence rate of “unexplained excision” resulting from inadequate preoperative preparation is significantly high [5]. Thus, complete tumor resection after thorough preoperative evaluation is critical to reduce postoperative recurrence risk [4]. In this case, evaluating the aorta and tumor invasion was important for complete tumor resection.

Although the angle of tumor contact, deformity, and loss of the mediastinal fat layer are criteria for assessing aortic invasion on CT and magnetic resonance imaging (MRI), these criteria are often overestimated [6]. Other tests used to assess invasion include four-dimensional CT, cine-MRI, intravascular ultrasound, and transesophageal echocardiography, which have sometimes been effective [7, 8]. Nonetheless, thoracoscopy is crucial to confirm whether the tumor invades the blood vessels. In this case, a contrast-enhanced CT scan showed an unclear arterial wall border in some areas, partly because of calcification. Based on thoracoscopic findings during a tumor biopsy by a previous clinician, the aortic invasion was determined to be high.

Given suspected aortic infiltration, two surgical options were considered: TAR with resection of the aortic aorta or debranching thoracic endovascular aortic repair (TEVAR) with stent-graft insertion at the expected infiltration site, followed by resection of the infiltrated aortic wall. The surgical method in combination with TEVAR is similar to that used for aortic invasion in lung cancer [5, 9]. Although the combined TEVAR procedure is less invasive than aortic arch replacement, it has disadvantages. A safety margin of 2–4 cm should be maintained centrally and peripherally for stent-graft insertion [2, 10]. In this case, imaging findings suggested tumor invasion of the left common carotid artery and the arch aorta from the peripheral brachiocephalic artery bifurcation to the bifurcation of the left common carotid artery. Considering the margin, the central side could have landed in Zone 0 of the aorta. Reports of debranching TEVAR in Zone 0 with an open chest indicate it is not a minimally invasive procedure and, thus, undesirable [11].

In a similar case, TEVAR was used to cover the aortic invasion site during tumor resection, leading to massive bleeding when the tumor and invasion site were dissected. In our case, a complete resection with a sufficient margin could have posed a similar bleeding risk. Another case report of postoperative aortic dissection in a patient with TEVAR also highlighted the procedure’s questionable safety aspects [12].

Since it was difficult to resect the tumor through a median sternotomy alone, this approach was thoroughly investigated. The left brachiocephalic vein and subclavian artery had to be secured more peripherally than usual, necessitating a good view of the left subclavian artery, so we performed a TMA. Nonetheless, this approach alone was insufficient to reach the tumor dorsally for adhesion dissection. Dorsal manipulation was safely and minimally invasively performed using thoracoscopy, allowing more efficient and safer dorsal adhesion dissection and pleurodesis [13]. The aforementioned procedure was carefully reviewed in a multidisciplinary preoperative conference with cardiovascular surgeons and anesthesiologists. We believe the elected procedure was performed reliably and safely to achieve a complete resection.

In this report, we describe our experience with a rare case of mediastinal tumor, characterized by a dedifferentiated liposarcoma that invaded the left common carotid artery, left subclavian artery, and distal aortic arch. Successful tumor resection was performed using TAR.

## Data Availability

Not applicable.

## Abbreviations

CT: Computed tomography
MRI: Magnetic resonance imaging
TAR: Total aortic replacement
TEVAR: Thoracic endovascular aortic repair
TMA: Transmanubrial approach
